# Multispectral Imaging Using Fluorescent Properties of Indocyanine Green and Methylene Blue in Colorectal Surgery—Initial Experience

**DOI:** 10.3390/jcm11020368

**Published:** 2022-01-13

**Authors:** Wojciech Polom, Marcin Migaczewski, Jaroslaw Skokowski, Maciej Swierblewski, Tomasz Cwalinski, Leszek Kalinowski, Michal Pedziwiatr, Marcin Matuszewski, Karol Polom

**Affiliations:** 1 Department of Urology, Faculty of Medicine, Medical University of Gdansk, 80-210 Gdansk, Poland; matmar@gumed.edu.pl; 2 2nd Department of General Surgery, Jagiellonian University Medical College, 31-501 Cracow, Poland; marcin.migaczewski@uj.edu.pl (M.M.); michal.pedziwiatr@uj.edu.pl (M.P.); 3 Department of Surgical Oncology, Faculty of Medicine, Medical University of Gdansk, 80-210 Gdansk, Poland; jaroslaw.skokowski@gumed.edu.pl (J.S.); maciejswierbel@tlen.pl (M.S.); cwalinski.tomasz@gmail.com (T.C.); karol.polom@gumed.edu.pl (K.P.); 4 Department of Medical Laboratory Diagnostics-Fahrenheit Biobank BBMRI.pl, Medical University of Gdansk, 80-210 Gdansk, Poland; leszek.kalinowski@gumed.edu.pl; 5 BioTechMed Centre/Department of Mechanics of Materials and Structures, University of Technology, 80-210 Gdansk, Poland

**Keywords:** colorectal surgery, ureter, methylene blue, indocyanine green

## Abstract

***Introduction*:** Image-guided surgery is becoming a new tool in colorectal surgery. Intraoperative visualisation of different structures using fluorophores helps during various steps of operations. In our report, we used two fluorophores—indocyanine green (ICG), and methylene blue (MB)—during different steps of colorectal surgery, using one camera system for two separate near-infrared wavelengths. ***Material and methods*:** Twelve patients who underwent complex open or laparoscopic colorectal surgeries were enrolled. Intravenous injections of MB and ICG at different time points were administered. Visualisation of intraoperative ureter position and fluorescent angiography for optimal anastomosis was performed. A retrospective analysis of patients treated in our departments during 2020 was performed, and data about ureter injury and anastomotic site complications were collected. ***Results*:** Intraoperative localisation of ureters with MB under fluorescent light was possible in 11 patients. The mean signal-to-background ratio was 1.58 ± 0.71. Fluorescent angiography before performing anastomosis using ICG was successful in all 12 patients, and none required a change in position of the planned colon resection for anastomosis. The median signal-to-background ratios was 1.25 (IQR: 1.22–1.89). Across both centres, iatrogenic injury of the ureter was found in 0.4% of cases, and complications associated with anastomosis was found in 5.5% of cases. ***Conclusions*:** Our study showed a substantial opportunity for using two different fluorophores in colorectal surgery, whereby the visualisation of one will not change the possible quantification analysis of the other. Using two separate dyes during one procedure may help in optimisation of the fluorescent properties of both dyes when using them for different applications. Visualisation of different structures by different fluorophores seems to be the future of image-guided surgery, and shows progress in optical technologies used in image-guided surgery.

## 1. Introduction

Image-guided surgery has become the new standard of intraoperative visualisation across countless applications [1,2,3]. Near-infrared imaging, which uses near-infrared light, requires fluorophores that are visible after excitation in a special imaging system [4]. These fluorophores may be used locally or systemically to obtain specific visualisation of structures. For a better intraoperative orientation, many camera systems have developed an overlaid colour facility that may help in the visualisation of required targets. Indocyanine green (ICG), methylene blue (MB), and 5-amino-levulinic acid are the only three fluorophores currently approved by the Federal Drug Administration and the European Medicines Agency. A separate camera system is required for individual fluorophores, as each must be excited and detected by a specific wavelength of light. ICG is a tricarbocyanine molecule that absorbs near-infrared light with a peak spectral absorption at a wavelength of 778 nm, and it emits back fluorescent light at 830 nm after excitation. Since the first publication of Kitai et al. showed the practical use of ICG fluorescence in the detection of breast cancer sentinel node biopsy, countless applications have been available for this fluorophore [5]. Intraoperative angiography, tumour detection, sentinel node biopsy, and ureter visualisation are just some of its practical applications in colorectal cancer surgery [6]. MB is a thiazine dye, a fluorophore that absorbs and emits back the light outside the near-infrared spectrum; thus, it should not be considered a pure near-infrared probe. Near-infrared fluorescence visualisation needs a fluorophore that presents a specific characterization: an excitation wavelength of 665 nm, with an emission of 686 nm [7]. This is one of the reasons why MB has less tissue penetration, and more autofluorescence of the background tissue. Additionally, caution should be used when using this dye in fluorescence in the presence of an operating theatre light, which may interfere with proper visualisation. Currently, five main domains of MB are available [3], namely the fluorescence visualisation of ureters, parathyroid glands, specific types of pancreatic tumours, breast cancer tumour margins, and sentinel node biopsy for breast cancer [3]. 

Our initial report presents the simultaneous use of ICG and MB with the same camera system for the visualisation of separate structures during colorectal cancer surgery. ICG was used in angiography for anastomotic perfusion evaluation, whereas MB was used in ureter detection. Currently, separate camera systems are used for both dyes’ fluorescent visualisation, and here, for the first time, both systems are used in the same camera to see ICG and MB separately during one operation. We compared the results with a retrospective analysis of cases operated in our centres in 2020, with specific reference to data associated with anastomotic site complications and ureter injury. 

## 2. Materials and Methods

This prospective preliminary study was conducted on patients with colorectal resection in the Surgical Oncology Department at the Medical University of Gdańsk, and II Department of General Surgery at Krakow Jagiellonian University, both in Poland. The institutional review board approved the study (NKBBN/259/2019). All patients were qualified for colorectal tumour resection after a multidisciplinary board review, and they signed informed consent forms. The selection was based on fluorescent system availability.

Patients with a confirmed allergy to ICG methylene blue or iodine substances, or at risk for serotonin syndrome or severe renal/liver disfunction were excluded from this study. The patients were not consecutive. Both open and laparoscopic operations were performed. 

The primary outcome was the feasibility of using one camera system with both fluorophores during colorectal surgery for ureter visualisation and colonic fluorescent angiography. The secondary outcome was the modification of the proximal colonic transection line according to the fluorescent indication. 

We performed a complete mesocolic or total mesorectal excision procedure with the ligation of the artery, and the vein-first approach with a high-tie ligation. 

During mobilisation of the colon on the right or left side, according to the tumour position, we examined the retroperitoneal space for ureter inspection. The inspection was not performed through the mesocolon. No additional tissue dissection was performed solely to visualise the ureter by naked eye or fluorescence. Additionally, we did not inspect the ureter on the other side. For ureter visualisation in open procedures, we dimmed the operating light, and relied on the light from the camera. This was not necessary in the case of laparoscopic procedures, since the light source was already within the required emission spectra.

We collected demographic and medical information from the patients, and we compared our preliminary data with a retrospective analysis of patients treated in our departments during 2020, a pandemic year that impacted the number of patients, and caused other organisational issues associated with local COVID-19 policies at our hospitals. We collected information about anastomotic site complications and ureter injury during the operations. 

### 2.1. Procedures for Intraoperative Real-Time Near-Infrared Fluorescence

#### 2.1.1. Methylene Blue

At the beginning of the operation, after anaesthesia, a dose of 0.5 mg/kg of MB after dilution in 5% glucose was injected intravenously. The dye (METIBLO, Laboratoires Sterop, Brussels, Belgium) had been purchased in 10 mg/1 mL vials. MB infusion was applied for about 20–30 min. Visualisation of the ureters was performed during preparation of the planes of complete mesocolic excision, and it was also carried out when there were doubts about ureter position within the inspected structures.

After MB administration and preparation of the area of interest during the colorectal surgery, we visualised the ureter in both open and laparoscopic approaches. First, we inspected the area under the white light, and again using fluorescence. Ureter visualisation under fluorescent light was considered as either ‘visible’ or ‘not visible’. The visibility was confirmed by the operator as the structure of a ureter. No additional tissue dissection or change in operation was performed to visualise the ureter. 

#### 2.1.2. Indocyanine Green 

After resection of the specimen, both anastomotic colorectal resection lines were carefully inspected. We injected 4 mL ICG (Pulsion, Munich, Germany) that had been diluted in distilled water to obtain a concentration of 2.5 mg/mL. This was done by rapid bolus injection to the peripheral vessel just before the fluorescent observation. The camera had already been prepared, and was ready for use prior to the ICG injection. Bowel perfusion was visualised, and subjectively evaluated by the surgical team in real time for about 3 min. 

We investigated the bowel perfusion in proximal and distal to the anastomotic area. The resection margin was marked in clinical visualisation first, and then confirmed or changed by adding fluorescent angiography. Afterwards, the anastomosis was performed. A re-resection of the bowel was performed in the case of insufficient fluorescent angiography of the anastomotic side. 

The Quest Artemis (Quest Medical Imaging, Middenmeer, The Netherlands) was designed and developed for open and minimally invasive, image-guided surgery using near-infrared fluorescence imaging (NIRF). The fluorescence imaging system was designed to visualise two types of fluorescent probes, cyanine 5.5 (Cy5.5) and ICG (or any other probe with similar fluorescent properties), which are not visible to the naked eye. For this study, the Cy5.5 mode was used to visualise the MB, with the tissue illuminated with a wavelength of 680 nm, and visualised at approximately 710 nm. During imaging, a colour image of the surgical field was visualised simultaneously with the NIRF to allow for surgical guidance. The ICG mode that we used to visualise the ICG was illuminated with a wavelength of 785 nm, and visualised at approximately 810 nm.

It was possible to observe the operation in one mode or with the screen divided into four parts, namely standard visibility, fluorescence visibility, and fluorescence that is superimposed on pseudocolour.

Visualisation of each dye was possible after simply switching the panel system between fluorophores. 

Each piece of recorded footage was re-analysed postoperatively, and the regions of interest (ROI) were marked to calculate signal-to-background ratio (SBR). The ROIs were drawn manually at the position of the fluorescent ureter, and at similar sized positions in the random background area. The same was performed during the colorectal angiography surgeries. 

## 3. Results

Twelve patients were enrolled in the study and analysed. Laparoscopic resection was performed in five cases, and open resection in seven cases. All patients had either total mesorectal excision or complete mesocolic excision with a high vascular ligation. In cases of high-grade adenoma and perforated tumour based on diverticulitis, low vascular ligations were performed. In cases of rectal and sigmoid cancer, a mobilisation of the splenic flexure was performed. The basic clinical and pathological characteristics of the patients are listed in Table 1.

**Figure 1:**
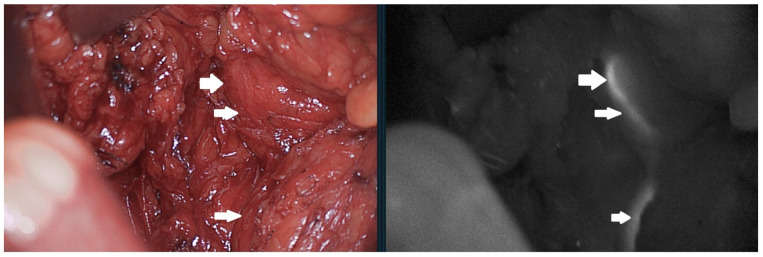
Ureter visualisation after methylene blue injection. Normal vision compared to fluorescent view. Arrows pointing the ureter in normal light and fluorescence.

Using fluorescence, we identified the ureter in 11 out of 12 patients (91.6%) (Figure 1). The unidentified ureter was not visualised during laparoscopic right hemicolectomy. 

We should mention that this was the first case performed in a laparoscopic setting. In one case after preoperative tumour perforation and abscess formation, the ureter and the left ovary were involved in the mass. We visualised the upper and lower parts of the ureter with fluorescence guidance, resected part of the ureter involved in the process to obtain free tumour margin, and reconstructed it intraoperatively. The position of the ureter was located more medially or laterally, according to possible expectations in two cases (18.2%). The mean signal-to-background ratio was 1.58 ± 0.71 (Shapiro—Wilk test *p* = 0.1327). All ureters were seen by the naked eye, but only those closest to the resection side. The goal for this study was not to look for fluorescent first visualisation—such a goal should be the aim for further research. 

Fluorescence angiography with ICG was obtained in all patients, without any adverse reaction. We did not change the surgical plan for anastomosis creation. We have not found any anastomotic leakage (AL) in the postoperative course of the analysed patients. 

The median signal-to-background ratios was 1.25 (IQR: 1.22–1.89) (Shapiro—Wilk test *p* = 0.0032). No postoperative mortality was recorded. For morbidity, we recorded only four complications of grade 1 and 2, according to the Clavien—Dindo classification [8]. 

We performed 104 colorectal resections at one centre. In one case, an iatrogenic injury of the ureter was found (0.96%), as well as three anastomotic site complications, namely two leakages of the anastomosis, and one necrosis of the permanent colostomy that required re-operations (2.8%). In the second centre, 132 colorectal operations were performed, and no ureter injury was found. In this group of patients, 10 (7.5%) anastomotic leakages were found, with 4 re-operations, and 6 cases of endoscopic vacuum-assisted closure that were successful for treating the complication. Across both centres, iatrogenic injury of the ureter was found in 0.4% of cases, and complications associated with anastomosis were found in 5.5% of cases. 

## 4. Discussion

Simultaneous use of ICG and MB with the same camera system for the visualisation of separate structures during colorectal cancer surgery is feasible. Fluorescent visualisation of ureters was found to be successful in 11 out of 12 patients (91.6%), and an ICG angiography was successful in all patents. Our primary outcome of the study was completed. The secondary outcome—the modification of the proximal colonic transection line according to the fluorescent indication—was not completed, as none of the patients required modification of the transection line based on ICG angiography. 

### 4.1. Fluorescent Visualisation of The Ureters

Ureters are structures which colorectal surgeons should be aware of during surgical resection. Iatrogenic injury of the ureter is not common, but it does lead to serious complications. In gynaecologic operations, the rate of injury might be as high as 1.5% of cases [9]. Visualisation of this structure is of great importance, especially in re-operations or after radiotherapy. Some preliminary reports about using light ureter catheters are available; however, this requires an additional invasive procedure, and may increase the risk of complications [10]. The first description of near-infrared fluorescence visualisation of ureters was proposed by Verbeek et al., where, in their study of a group of 12 patients, both ureters were seen after intravenous injection of MB [11]. The authors used three different doses of MB (0.25 mg/kg, 0.5 mg/kg, and 1 mg/kg), and the difference was only seen at the time of exposure. The other publication on this topic is based on 10 patients by Al Taher et al., who visualised both ureters of 5 of the patients under fluorescence [12]. In a study based on a much larger group of patients, Barnes et al. identified 64 out of 69 ureters during colorectal surgery in 40 patients [13]. Since 14 ureters were not seen in visible light, the help of fluorescence made an impact in this group of patients. Additionally, 50 ureters were seen under visible, as well as fluorescent, light, but 14 of them were recognised more quickly using near-infrared visualisation. The authors highlighted that in 14.5% of cases, the ureter was identified in a different localisation than expected. They also used different concentrations of methylene blue (0.25 mg/kg, 0.5 mg/kg, 0.75 mg/kg, and 1 mg/kg). The highest mean SBR was seen using the 0.75 mg/kg dose (mean = 5.29, SD = 2.72, 95% CI 4.84–5.75), and the lowest when using 1 mg/kg (mean = 3.66, SD = 1.89, 95% CI 3.37–3.39). Contrary results were published by Yeung et al., whereby a concentration of 1 mg/kg gave the strongest signal [14]. We should underline the fact that not only MB, but also other dyes, are used for visualising ureters, such as ICG. After retrograde injection to the ureter through ureteral catheters after ICG injection, an immediate visualisation is possible [15,16,17]. In a group of 30 pelvic surgeries, Mandorva et al. observed the fluorescence of ureters in all cases, and the fluorescence was visible until the end of the longest operation (about 4 h). In 33% of patients, the fluorescent light helped in the localisation of ureters prior to naked-eye inspection. Additionally, two other experimental dyes are available: IRDye 800BK and cRGD-ZW800-1. Both are being investigated in ongoing trials (trial numbers 2017-001954-32 and NCT03387410). In a review by Slooter et al., another six dyes (CW 800-CA, Fluorescein, Liposomal ICG, Genhance 750, UL-766, Ureter Glow) were considered to be valuable options for ureter visualisation; however, only preliminary results are available [18]. In our study, we visualised the ureter in 91.6% of cases, and our retrospective analysis showed that injury of the ureter was very rare, although still possible. In difficult cases, it seems that the help of fluorescent visualisation may be of great importance, especially in difficult cases, and during the training of young surgeons. 

We should mention the limitations of using MB. First, is the impairment of renal function, as MB is excreted with urine by the kidneys. Another limitation is that MB use is restricted to patients who convert this dye into non-fluorescing leucomethylene blue. Allergic reactions, quenching effect, usage of the diuretics, and low penetration through fatty tissue are the most common limitations described in the literature. More details are available in our recently published review about MB usage in surgery [3]. The limitations of ICG usage are also described in our other publication [2].

### 4.2. Tissue Perfusion for Anastomotic Creation 

AL is among the most difficult postoperative complications to treat in colorectal surgery, reaching as many as 20% of cases in some series [19]. This complication is multifactorial, and factors such as host genetics, gut microbiome, inflammation process, and reaction of the immune system may play an important role in it [20]. Another important factor associated with anastomotic healing is inadequate anastomotic vascular perfusion, which might be visualised using ICG as a fluorophore during surgery. After intravenous injection, an intraoperative fluorescence angiography is performed to visualise a real-time perfusion of the resection margin. In the multi-centre PILLAR II trial, such fluorescence perfusion changed the intraoperative approach in 11 patients (7.9%) [21]. Inadequate serosal evaluation was found in nine patients, and inadequate postanastomotic mucosal evaluation in two patients; importantly, none of them developed postanastomotic leakage. In a systematic review and network meta-analysis of a group of patients who underwent ICG angiography, a significant difference in AL was found in favour of ICG (RR 0.44; Crl 0.14–0.87) compared with the control group [22]. However, we should underline the fact that AL may still occur even after ICG angiography. In the publication of Otero-Piñeiro et al., the AL dropped dramatically after ICG implementation, but was still present in some patients (11.3% vs. 2.5%) [23]. 

In our series, no postoperative AL was found. We did not change the site of anastomosis because of poor fluorescent angiography. The SBR of our patients was median 1.27. In the retrospective series, AL occurred in 5.5% of cases. 

Other recently published topics related to ICG usage in colorectal surgery show that not only the surgeon’s eye, but also quantification of bowel perfusion, might be used to improve the quality of fluorescence angiography. In the publication of Wada et al., maximum intensity ICG was found to be a predictor of AL [24]. In the publication by Hayami et al., the time from injection of ICG to the beginning of fluorescence was significantly longer in the group of patients who developed AL [25]. In the publication by Park et al., the first implementation of artificial intelligence for real-time microcirculation analysis using fluorescent ICG properties showed that it was more accurate and consistent than currently used methods [26]. Future research is awaited to use signal-to-background ratio as a tool in quantification of the ICG fluorescence. In our series, the analysis showed that such a tool might be used also in fluorescent angiography, as well as in other research based on novel dyes [27,28].

### 4.3. Colorectal Multichannel Visualisation 

Recently, a publication presenting the first usage of multi-wavelength fluorescence imaging in colorectal surgery has showed the possibility of using a standard Firefly system to visualise lymphatic vessels with fluorescein and lymph nodes with ICG [29]. For similar visualisation of both fluorophores, they had to adjust fluorescein concentration. Another group showed that the robotic Firefly Si system, with some modifications, was able to visualise far red dye (Cy5) [30]. Both publications revealed that some technical changes in Firefly robotic platforms might lead to a multispectral camera system for Da Vinci robotic systems. Our publication, for the first time, shows that, using a single camera system, we are able to see the location of the ureter, and that this may increase the safety of the operation, as well as help in finding the optimal anastomotic site following ICG angiography. Moreover, ICG is also used in the visualisation of ureters or for sentinel node biopsy in colorectal surgery, which may interfere with fluorescent visualisation for further angiography. This is of particular importance in cases of using quantification of angiography and visualising other structures where the same dye may interfere with a correct calculation. Using two separate dyes during one procedure may help in the optimisation of the fluorescent properties of both dyes so that they can be used for different applications. Such a possibility also plays an important role, especially, in the age of new fluorophores that will show a variety of possibilities for targeted and non-targeted fluorescence image-guided surgery.

Complete oncological resection and preservation of important structures during colorectal surgery are key points to obtain optimal results during and after an operation. Recent advancements in near-infrared fluorescent-guided surgery have helped us to see more and gain a better understanding of many factors that may help surgeons decrease the morbidity and mortality of their patients. Different fluorescent emissions may help in multichannel visualisation of multi-wavelength (also called multispectral, multiplexing, or multicolour) surgical visualisation. Using this technique, different anatomical structures can be seen, including tumours, ureters, lymphatic vessels, and nerves. Previous publications have shown that, under microscope multiplexing, visualisation is a standard way of differentiating important structures. However, only in recent years have fluorescein and ICG both been used in robot-assisted laparoscopic operations [31,32].

Our initial report presents the first application of MB and ICG when used in the same camera system during the same operation, for safer surgery and better outcomes. The usage of ICG and MB, based on their safety profiles, is the best example of easily accessible fluorophores that may help in obtaining better results in surgery. Other fluorophores, and especially targeted ones, will probably soon be able to change the way we see and differentiate structures during surgery. Another approach is integrated multicolour fluorescence visualisation, instead of the sequential one presented in our study. Here, we did not need to see both fluorophores at once, but this might be an interesting step forward in multispectral imaging. However, in the Firefly system that is most commonly used during robotic surgery, a white light mode is used, and the fluorescence mode is only used when required, often just for the confirmation of already exposed structures. The future will show us the direction in which intraoperating visualisation will follow. 

Another important issue to discuss is the possible importance of new tracers, such as targeted fluorophores, in tumour visualisation. A fluorescent antibody that targets carcinoembryonic antigen (SGM-101) has been developed, and some preliminary results have been delivered [33]. An improvement in peritoneal carcinomatosis index during cytoreductive surgery for colorectal peritoneal metastases was found [34]. Another novel fluorescent peptide, cRGD-ZW800-1, was investigated and found to have clinical utility in colorectal surgery [27]. After intravenous injection, the fluorescent tumour was visible through a full-thickness wall. It is possible, in a multispectral way, that we can use different fluorophores which will be responsible for different tasks during surgery.

The biggest limitation of our study is the small number of patients. Undoubtedly, after the first proof of concept research, the next studies will answer the other important issues associated with multichannel visualisation in colorectal surgery. 

## 5. Conclusions

Our initial report presents an application of one camera system for two different fluorophores during surgery, leading to a safer operation and a better outcome. The visualisation of ureters, and the decreased rate of postoperative AL are both responsible for better outcomes in colorectal surgery. Our material shows that both applications may find a place in colorectal surgery that is done with the same camera during different points of the operation to help decrease the complication rate. This is also supported by the results of our retrospective analysis. Possible multi-fluorophore usage during surgery for specific steps during operations seems to be the future of image-guided surgery and progress in optical technologies used in image guided-surgery. Further research is justified.

## Figures and Tables

**Table 1:** Clinicopathological data of patients.

	Number of Patients
Patient (n)	12
Age, y (mean)	68.3 ± 6.75
Sex (male, female)	9:3 (75%:25%)
Smoking	
Yes	6 (50%)
No	6 (50%)
Diabetes	
Yes	4 (33.3%)
No	8 (66.7%)
BMI mean	27 ± 5.03
Neoadjuvant chemotherapy	
Yes	4 (33.3%)
No	8 (66.7%)
Neoadjuvant	
radiotherapy	
Yes	3 (25%)
No	9 (75%)
Type of intervention	
Right hemicolectomy	2 (16.7%)
Rectum anterior resection	5 (41.7%)
Rectum anterior resection + ileostomy	2 (16.7%)
Hartmann operation	1 (8.3%)
Abdominal sacral resection	1 (8.3%)
pT	
0	2 (16.7%)
1	1 (8.3%)
2	3 (25%)
3	4 (33.3%)
4	2 (16.7%)
pN	
0	6 (50%)
1	5 (41.7%)
2	1 (8.3%)
M	
M0	12 (100%)
M1	0 (0%)

## Data Availability

The data presented in this study are available on request from the corresponding author.
